# Health effects of exposure to radon: implications of the radon bed mattress incident in Korea

**DOI:** 10.4178/epih.e2019004

**Published:** 2019-02-12

**Authors:** Songwon Seo, Wi-Ho Ha, Jin-Kyu Kang, Dalnim Lee, Soojin Park, Tae-Eun Kwon, Young Woo Jin

**Affiliations:** National Radiation Emergency Medical Center, Korea Institute of Radiological and Medical Sciences, Seoul, Korea

**Keywords:** Radon, Exposure, Neoplasms, Carcinogens, Risk

## Abstract

Radon is a naturally occurring radioactive material formed by the slow decay of uranium and thorium found in the earth’s crust or construction materials. Internal exposure to radon accounts for about half of the natural background radiation dose to which humans are exposed annually. Radon is a carcinogen and is the second leading cause of lung cancer following smoking. An association between radon and lung cancer has been consistently reported in epidemiological studies on mine workers and the general population with indoor radon exposure. However, associations have not been clearly established between radon and other diseases, such as leukemia and thyroid cancer. Radiation doses are assessed by applying specific dose conversion coefficients according to the source (e.g., radon or thoron) and form of exposure (e.g., internal or external). However, regardless of the source or form of exposure, the effects of a given estimated dose on human health are identical, assuming that individuals have the same sensitivity to radiation. Recently, radiation exceeding the annual dose limit of the general population (1 mSv/yr) was detected in bed mattresses produced by D company due to the use of a monazite-based anion powder containing uranium and thorium. This has sparked concerns about the health hazards for mattress users caused by radiation exposure. In light of this event, this study presents scientific information about the assessment of radon and thoron exposure and its human implications for human health, which have emerged as a recent topic of interest and debate in society.

## INTRODUCTION

In South Korea (hereafter Korea), the dose of natural background radiation exposure among the general population is about 3 mSv/yr, half of which is caused by internal exposure to radon [1]. Of the numerous radioactive isotopes of radon, radon (^222^Rn) and thoron (^220^Rn) are those mostly present in the natural environment. Thoron has a shorter half-life than radon and does not significantly contribute to natural background radiation exposure; therefore, it is not subject to management or regulation.

Because radon is a noble gas with a relatively short half-life, it is maintained in low concentrations in well-ventilated environments. However, it can be present in high concentrations in closed spaces, and it may cause health hazards, such as lung cancer.

Recently, using a commercially available radon detector, a homemaker unintentionally discovered that the radon and thoron concentrations in a bed mattress produced by D company exceeded the indoor limit [2]. After the media conveyed this news, concerns emerged concerning internal exposure to radon and thoron among users of the said mattress as a serious social issue. The problematic mattress contained monazite, a source of radon and thoron. In particular, because of the high thorium content in the monazite, the thoron concentration was found to be 10 times higher than that of radon, and many of the mattresses were found to emit radiation in excess of the annual dose limit of 1 mSv designated by the Act on Protective Action Guidelines Against Radiation in the Natural Environment. Although the indoor atmospheric thoron concentration is generally predicted to be insignificant due to its short half-life, the problem with bed mattresses containing thoron is that they could be a major source of radiation exposure from thoron because the mattresses come into close contact with users’ respiratory organs.

**Figure 1.**
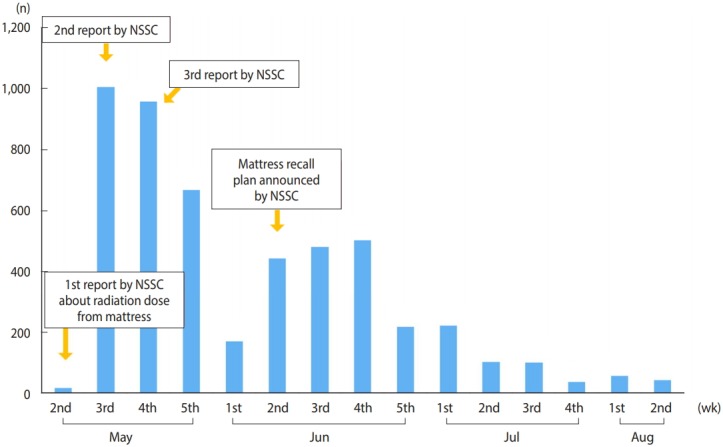
Number of weekly telephone calls for counseling at the Korea Institute of Radiological and Medical Sciences concerning the health effects of using radon bed mattresses. NSSC, Nuclear Safety and Security Commission.

To date, the risks of radon and thoron exposure have not been a topic of interest, unlike artificial radiation related to nuclear power plants and nuclear facilities, and the impact of thoron on human health remains unknown. However, following media coverage and the announcement made by the Nuclear Safety and Security Commission (NSSC) about the radioactive material-containing bed mattresses made by D company, social interest and concerns regarding the health hazards created by radon and thoron exposure have risen, particularly among mattress users (Figure 1). In response, the government and relevant public agencies have begun to provide accurate information about radon exposure via various communication channels, including phone calls, e-mails, social media, face-to-face consultations, and information sessions. However, at the same time, erroneous information swept the Internet and social media, causing confusion among mattress users. Therefore, this article aims to provide scientific information regarding the assessment of radon and thoron exposure and its implications for human health, which have emerged a topic of interest and debate in society. Most epidemiological studies on the effects of radon and thoron on human health have focused on radon exposure at work sites and in indoor living environments, and too few studies have been conducted to compare the effects of radon and thoron independently. Thus, this study primarily focuses on the effects of radon exposure on human health. However, considering that both radon and thoron emit alpha particles, it is predicted that exposure to an equal dose to either would result in similar effects on human health.

## DOSE ASSESSMENT OF INTERNAL EXPOSURE TO RADON AND THORON

Radon (^222^Rn, half-life: 3.8 days) and thoron (^220^Rn, half-life: 56 seconds), which are categorized as naturally occurring radioactive materials, are produced when uranium (^238^U) and thorium (^232^Th) present in the earth’s crust or construction materials slowly decay [3]. Because internal exposure to radon accounts for about half of the dose of natural background radiation exposure, the Korea Institute of Nuclear Safety and National Institute of Environmental Research of the Ministry of Environment monitor and manage radon concentrations in indoor and outdoor environments [4]. Regardless of the potential importance of thoron, radon is the major source of background radiation exposure because of thoron’s short half-life. However, processed products with high concentrations of monazite, a source of thorium, which produces thoron, may cause higher internal exposure to thoron than to radon.

In general, the internal dose is assessed by using intake and internal dose conversion coefficients called dose per unit intake (DPUI) of a particular radionuclide. The dose conversion coefficients are provided by the International Commission on Radiological Protection (ICRP), and selecting the correct dose conversion coefficient is important, as it differs according to the type of radionuclide, size of particles, and absorption rate into body fluids by pathway of intake. Intake of radionuclides can generally be assessed using bioassay measurements, such as whole-body counting or urine measurements, or by considering the airborne activity concentration, breathing rate, and length of work. However, because radon and thoron are noble gas-type radionuclides with a short half-life, and their progenies, which account for most of the exposed dose in the lungs, are short-lived, they rapidly decay before the particles are transferred into the blood or to other organs. Hence, general bioassay measurements cannot be used to assess the dose of radon and thoron. Instead, internal exposure to radon and thoron is assessed and managed using the activity concentration of radon and thoron in the atmosphere.

The use of dose conversion coefficients differentiates the assessment of internal exposure to radon and thoron from those of other radionuclides. As previously mentioned, the DPUI is used for other types of radionuclides, but the ICRP recommends the use of other dose conversion coefficients for radon and thoron, including the effective dose per working level month (WLM). The WLM is a special unit that expresses the cumulative exposure to radon, thoron, and their progeny for a working month (170 hours) in an environment that emits potential alpha energy (PAE) from each radionuclide progeny in 1 L of air. In general, the equilibrium factor (F), which is the ratio of the concentration of radon/thoron in a non-equilibrium state to the concentration of radon/thoron and progeny in an equilibrium state, must be taken into consideration when using concentrations of radon and thoron in the atmosphere. The equations for computing the equilibrium equivalent concentration (EEC) and the internal dose using the EEC are as follows [3,5].

(1) EEC =concentration of radon/thoron in the atmosphere×equilibrium factorInternal dose= EEC× dose conversion

(2 coefficient× duration of exposure

Another factor that complicates efforts to quantify and manage internal exposure to radon and thoron and its consequent risks is the fact that dose assessment of radon exposure can be performed using either the epidemiological method or the dosimetric method. ICRP 65 [6] uses an epidemiological method, where the dose conversion coefficient to effective dose per unit exposure is computed by comparing the cancer risk factor per radon exposure to the risk factor per unit effective dose. This is known as the dose conversion convention, and it differs from the dosimetric assessments of other radionuclides. However, ICRP 115 [7] re-assessed the dose coefficients by considering not only the existing epidemiological data on mine workers but also study data on living spaces, which resulted in the previous effective dose coefficients to increase more than twofold. Finally, based on a comprehensive review of existing studies, ICRP 126 [3] concluded that the results of dose assessments using epidemiological data are similar to those using dosimetric models. Therefore, the ICRP decided to use the dose conversion coefficients from the dosimetric model, as is the case for other radionuclides. With the dosimetric method, the dose conversion coefficients to radon and its progeny are assessed using a biokinetic model and a dosimetric model, such as the human respiratory tract model (HRTM). A benefit of this method is that internal dose exposure in organs other than the lungs can be assessed. Table 1 shows the dose conversion coefficients for radon and thoron provided by the ICRP [6-8].

Computations using dosimetric models, such as the HRTM, revealed that the equivalent dose to the lung per unit exposure is relatively insensitive to age. For example, the dose of radon exposure in the lungs between adults and children differs only by 10%. This is because the lower dose caused by the smaller intake in children due to their lower breathing rate is offset by the increase in the dose caused by the smaller target cell mass. The differences in the dose per unit exposure of thoron across age groups have also been found to be less than 10%. Although some online media, such as blogs, news, and the former Agency for Toxic Substances and Disease Registry website (https://www.atsdr.cdc.gov/), state that the effects of radon exposure are more intense in children than in adults, evidence supporting this argument remains scarce.

When assessing internal exposure to radon and thoron, variability and uncertainty of the factors taken into consideration must be addressed. First, concentrations of radon/thoron in the atmosphere vary according to the point and period of measurement. Therefore, it is important to choose a reasonable representative value. Further, the reliability of the measurement device should be established, and the radon and thoron concentrations should be separately measured. The F for radon and thoron in indoor environments are generally 0.40 and 0.03, respectively, but the F for thoron may vary widely even in a small space because of its short half-life. Thus, the ICRP suggests that directly managing lead (^212^Pb), the thoron progeny with the highest contribution to PAE, is more appropriate than using the concentration of thoron in the atmosphere. In addition, the computed dose conversion coefficient may differ according to the size distribution of aerosols, air inhalation rate, the progeny’s aerosol attachment rate, lung deposition and blood absorption rates, and the HRTM. In the initial assessment, using the dose conversion coefficient provided by the ICRP that reflects the latest study results would be desirable, and when necessary, the various factors mentioned above should be considered to obtain a more detailed individual assessment of internal exposure.

## HEALTH EFFECTS OF EXPOSURE TO RADON

Radon is a carcinogen designated by the World Health Organization and is the second-leading cause of lung cancer following smoking. Approximately 3-14% of all lung cancers worldwide are estimated to be caused by radon exposure, and an association between radon and lung cancer has been consistently reported in studies investigating work environments of mine workers and indoor radon exposure. However, associations between radon and other diseases have yet to be established due to inconsistent study results and low biological relevancy. One reason is that radon is unlikely to penetrate organs other than the lungs because of the low penetrability of the alpha particles that are emitted during the decay of radon.

### Radon exposure and lung cancer

Although a rise in mortality caused by respiratory diseases among mine workers was observed in Europe in the 16th century, it was only in the 1950s that radon exposure was pinpointed as a relevant mechanism. In light of subsequent studies that established that mine workers exposed to radon had a higher risk of lung cancer, the International Agency for Research on Cancer classified radon as a human carcinogen in 1988.

Epidemiological studies on mine workers have generally adopted a cohort study design, which is best suited for establishing the causality between exposure and disease. In addition to studies conducted independently in different countries, including Germany and France, the Committee on the Biological Effects of Ionizing Radiation and the United Nations Scientific Committee on the Effects of Atomic Radiation comprehensively assessed individual studies on mine workers (Table 2) [9-16]. Their major findings showed that the excessive relative risk per increase of 100 WLM ranged from 0.55 to 0.59, indicating that increasing amounts of radon exposure were associated with an elevated risk for lung cancer. Furthermore, some studies also considered external exposure and quartz exposure in addition to radon exposure, and other studies considered smoking as the leading cause of lung cancer. Nonetheless, to summarize, studies have found that occupational exposure to radon increased the risk of lung cancer.

Findings showing radon exposure in the work environment among mine workers and an increased lung cancer risk among them sparked interest in the risk of radon exposure in indoor living environments. In several countries, including Korea, the USA, and European countries, ecological studies and case-control studies were conducted (Table 3) [17-24]. Because of the difficulty of directly assessing the exposure dose among individual study participants, these studies assessed associations between indoor radon exposure and lung cancer using the mean radon concentration per area. A case-control study in 13 European countries [17] found that the risk of lung cancer mortality increased by 16.0% with each 100 Bq/m^3^ increase in the indoor radon concentration, and the dose-response relationship supported a linear model without a threshold dose. When smokers and non-smokers were separated, the lifetime (75-year lifespan assumption) cumulative lung cancer risk in non-smokers was 0.4% at 0 Bq/m^3^ and 0.7% at 400 Bq/m^3^, while that among smokers was 10.0% at 0 Bq/m^3^ and 16.0% at 400 Bq/m^3^. In other words, the increase in lung cancer risk with increased radon exposure was not markedly different according to smoking status. However, the baseline risk for lung cancer was substantially higher among smokers; therefore, the absolute risk for lung cancer caused by radon exposure was found to be much higher (about 25 times higher) among smokers than among non-smokers. Similar results were found in subsequent studies, but the results were not as consistent as those on occupational exposure among mine workers. This may be attributable to the limitations of the nature of ecological studies and case-control study designs and the relatively low level of radon exposure compared to the amount of occupational exposure.

### Other diseases

Although the increased risk of lung cancer due to radon exposure is a well-known scientific fact, associations between radon exposure and other diseases have not been established. In particular, many studies have investigated the effects of radon exposure on leukemia. Some studies that used an ecological study design reported such an association, but the results of ecological studies can only be used for reference and are not conclusive because other confounding factors that may cause lung cancer cannot be eliminated. Thus, these results only imply the need for additional studies on the matter. A review article on radon and pediatric leukemia [25] observed a statistical significance in 3 of 7 case-control studies, but 1 of these studies found a negative association between acute lymphoblastic leukemia and radon exposure. The 4 remaining studies did not report statistically significant data. Therefore, the results on the association between radon exposure and leukemia are inconsistent across studies. Furthermore, it is physiologically unlikely that radon and its progeny would cause leukemia due to the nature of their pharmacokinetics; this possibility is not accepted as an established scientific theory, and additional studies are needed. A recent review article on central nervous system (CNS) tumors caused by radon exposure [26] reviewed 18 studies on underground mine workers. They found that some studies reported the possibility of CNS tumor development in response to radon exposure. However, it is difficult to conclude that radon exposure induces CNS tumors based on those results because the nature of the underground mine environment makes it uncertain whether CNS tumors were caused by radon exposure or by external exposure in the mines. For similar reasons, it has proven difficult to establish associations between radon exposure and thyroid cancer, skin cancer, kidney cancer, and heart disease.

## CONCLUSION

The assessment of risk for lung cancer from radon and thoron exposure must be preceded by accurate dose assessment, and a more precise risk assessment model is required. Although the results on the risk of occupational exposure to radon among mine workers are fairly consistent, the results on the effects of indoor radon exposure on human health widely vary across countries. Further, although existing studies have reported that there are minimal differences in the risk per dose according to smoking status, age, and gender, it remains challenging to establish a sound scientific consensus due to the scarcity of relevant data. Therefore, the uncertainty of risk assessment models should be ameliorated through additional studies. In particularly, as there are only a handful of cases of thoron exposure worldwide, relevant data should be acquired from international joint studies to be used for dose and health impact assessments.

The recent incident of radon and thoron exposure from the bed mattresses produced by D company is definitely an unnecessary accident, considering that the amount of exposure exceeded the dose limit for the general population. Based on the level of radiation exposure reported by the government and the results of studies worldwide, it is predicted that there would be no acute effects of radiation exposure among mattress users. However, we cannot definitively conclude that there are no concerns for the long-term risk of lung cancer. Epidemiological studies of users of the bed mattresses produced by D company can play an important role in not only evaluating health risks among the users, but also improving our knowledge of the health effects of radon and thoron. Therefore, a more meticulous review should be conducted on the need to conduct health impact assessments through epidemiological studies, including dose assessments that consider the actual use of the mattress, such as sleep duration and posture.

About half of the natural background radiation exposure among people in Korea is from internal exposure to radon, which is about twofold higher than the average exposure level worldwide. However, social awareness on the risk of radon exposure remains low. Hence, society must learn from the recent radon mattress incident and step up our efforts to lower exposure and promote health by ameliorating relevant policies and promoting relevant education and research. Furthermore, because the risk of lung cancer from radon exposure is significantly higher among smokers, quitting smoking is essential to lower the disease burden caused by radon.

## Figures and Tables

**Table 1.** Dose conversion factors for radon/thoron effective dose based on the dose conversion convention and dosimetric model

Category [Ref]	Dose conversion factor (mSv/WLM)	
	Radon^1^	Thoron^2^
ICRP 65 (dose conversion convention using past epidemiologic data) [6]	5 (adults)	-
	4 (all ages)	
ICRP 115 (dose conversion convention using additional epidemiologic data) [7]	12 (adults)	-
	9 (all ages)	
ICRP 137 (assessment using dosimetric model) [8]	20 (indoor work site)	5.6 (indoor work site)
	11 (mines)	4.8 (mines)
	23 (tourist caves)	
ICRP 137 (recommended value reflecting epidemiological data and dosimetric model) [8]	10 (e.g., underground mines, inside buildings)	5.0 (e.g., underground mines, inside buildings)

ICRP, International Commission on Radiological Protection; WLM, working level month; F, equilibrium factor; EEC, equilibrium equivalent concentration.

1 WLM for radon: 1 WLM=(6.37×10^5^/F) Bq-h/m^3^ (applying radon concentration).

2 WLM for thoron: 1 WLM=(4.68×10^4^) Bq-h/m^3^ (applying EEC of thoron).

**Table 2.** Risk of lung cancer caused by occupational exposure to radon (mine workers)

Country	Sample size	Follow-up period	M/I	No. of lung cancer cases	ERR/100 WLM (95% CI)
Germany [9]	58,974	1946-2013	M	3,942	2.31 (1.20, 4.13)
Czech [10]	9,978	1952-2010	M	1,141	0.97 (0.74, 1.27)
France [11]	5,400	1946-2007	M	211	0.73 (0.32, 1.33)
Canada [12]	17,660	1950-1999	M	618	0.55 (0.37, 0.78)
Canada [12]	16,770	1969-1999	I	626	0.55 (0.37, 0.81)
Canada [13]	1,742	1950-2001	M	191	0.43 (0.23, 0.62)
Sweden [14]	5,486	1958-2000	I	122	2.20 (0.23, 3.77)
Worldwide (7 countries) [15]	60,606	1943-1991	M	2,674	0.55 (0.27, 1.13)
Worldwide (6 countries) [16]	125,627	1946-2001	M	5,477	0.59 (0.35, 1.00)

M, mortality; I, incidence; ERR, excess relative risk (risk of additional disease incidence [or mortality] caused by exposure); WLM, working level months; CI, confidence interval.

**Table 3.** Risk of lung cancer due to indoor radon exposure

Country	Study design [Ref]	Sample size (n)		Follow-up period	Assessment indices^1^	Gender or smoking status	Risk (95% CI)
		Case	Control				
Korea	Ecological [18]	Administrative districts: 234		1999-2008	RR	Men	1.10 (1.00, 1.22)
		Population (average): 172,857					
		Lung cancer cases (average): 679				Women	1.10 (0.90, 1.22)
USA	Case-control [19]	1,474	1,811	1989-1993	ERR	Total	0.00 (-0.21, 0.21)
		846	1,029			Men	0.01 (-0.29, 0.27)
		628	789			Women	0.06 (-0.30, 0.43)
		205	484			Non-smokers	0.14 (-0.47, 0.75)
	Case-control [20]	651	740	1989-1992	EOR	Total	0.05 (-0.14, 0.56)
		349	392			Men	-0.13 (-0.30, 0.44)
		302	348			Women	0.29 (-0.12, 1.70)
	Case-control [21]	200	397	1990-1999	aOR	Total	0.04 (-0.20, 0.35)
Czech	Case-control [22]	370	1,399	1960-2010	ERR	Total	0.64 (0.11, 10.5)^2^
		58	670			Non-smokers	0.73 (0.02, 1.90)^2^
		312	729			Smokers	0.14 (0.02, 0.30)^2,3^
France	Ecological [23]	Population: 47,923,391		2008-2012	ERR	Men	0.25 (0.09, 0.48)
		Lung cancer cases: 29,690				Women	0.04 (-0.06, 0.22)
European (9 countries)	Case-control [17]	7,148	14,208	-	RR	Total	1.16 (1.05, 1.31)
Worldwide (13 countries)	Meta-analysis[24]	13,380	22,102	-	OR (highest vs. lowest)	Total (22 studies)	1.29 (1.10, 1.51)
		10,345	18,233		OR	Total (17 studies)	1.07 (1.04, 1.10)

RR, relative risk; ERR, excess relative risk; EOR, excess odds ratio; aOR, adjusted odds ratio (adjustment for smoking, duration of residence, level of education, income level, and duration of occupational carcinogen exposure); OR, odds ratio; CI, confidence interval.

1 All risks refer to risk caused by an increase in radon exposure by 100 Bq/m^3^.

2 90% CI.

3 Smokers who smoked 15-24 cigarettes a day.
